# Identification of Human Fibroblast Cell Lines as a Feeder Layer for Human Corneal Epithelial Regeneration

**DOI:** 10.1371/journal.pone.0038825

**Published:** 2012-06-18

**Authors:** Rong Lu, Fang Bian, Jing Lin, Zhitao Su, Yangluowa Qu, Stephen C. Pflugfelder, De-Quan Li

**Affiliations:** 1 Zhongshan Ophthalmic Center, State Key Laboratory of Ophthalmology, Sun Yat-Sen University, Guangzhou, China; 2 Ocular Surface Center, Cullen Eye Institute, Department of Ophthalmology, Baylor College of Medicine, Houston, Texas, United States of America; 3 Department of Ophthalmology, Union Hospital of Tongji Medical College, Huazhong Science and Technology University, Wuhan, China; 4 Department of Ophthalmology, The Affiliated Hospital of Qingdao University Medical College, Qingdao, China; Instituto Butantan, Brazil

## Abstract

There is a great interest in using epithelium generated in vitro for tissue bioengineering. Mouse 3T3 fibroblasts have been used as a feeder layer to cultivate human epithelia including corneal epithelial cells for more than 3 decades. To avoid the use of xeno-components, we evaluated human fibroblasts as an alternative feeder supporting human corneal epithelial regeneration. Five human fibroblast cell lines were used for evaluation with mouse 3T3 fibroblasts as a control. Human epithelial cells isolated from fresh corneal limbal tissue were seeded on these feeders. Colony forming efficiency (CFE) and cell growth capacity were evaluated on days 5–14. The phenotype of the regenerated epithelia was evaluated by morphology and immunostaining with epithelial markers. cDNA microarray was used to analyze the gene expression profile of the supportive human fibroblasts. Among 5 strains of human fibroblasts evaluated, two newborn foreskin fibroblast cell lines, Hs68 and CCD1112Sk, were identified to strongly support human corneal epithelial growth. Tested for 10 passages, these fibroblasts continually showed a comparative efficiency to the 3T3 feeder layer for CFE and growth capacity of human corneal epithelial cells. Limbal epithelial cells seeded at 1×10^4^ in a 35-mm dish (9.6 cm^2^) grew to confluence (about 1.87–2.41×10^6^ cells) in 12–14 days, representing 187–241 fold expansion with over 7–8 doublings on these human feeders. The regenerated epithelia expressed K3, K12, connexin 43, p63, EGFR and integrin β1, resembling the phenotype of human corneal epithelium. DNA microarray revealed 3 up-regulated and 10 down-regulated genes, which may be involved in the functions of human fibroblast feeders. These findings demonstrate that commercial human fibroblast cell lines support human corneal epithelial regeneration, and have potential use in tissue bioengineering for corneal reconstruction.

## Introduction

Ocular surface diseases with corneal epithelial stem cell deficiency are sight threatening and often cause blindness [1]. There is great interest in using corneal epithelium generated in vitro to treat these conditions and restore vision [2]–[7]. This requires development of culture technique that mimics the in vivo environment. Mesenchymal-epithelial interactions play an essential role in organogenesis and tissue regeneration at both embryonic [8], [9] and adult stages [10]–[12], and have been applied to in vitro cell culture systems for growing epithelial cells that were difficult to be cultivated.

The single-cell clonal growth was first achieved for keratinocytes by co-culturing with mouse 3T3 fibroblasts as a feeder layer [13], [14]. This 3T3 fibroblast co-culture system has been proven to be the most powerful system for human epithelial cultivation [7], [15]–[17], including human ocular surface epithelia [18]–[21], over the past 3 decades. Based on our observation, the proliferative rate and regenerative capacity of human corneal epithelial cells on the 3T3 fibroblast feeder layer were 10–50 times higher than that on explant cultures [22]. As few as 200–1000 limbal epithelial cells, which can be obtained from as small as 0.5 mm^2^ of limbal biopsy, could regenerate a whole human corneal epithelium in 2 weeks with this co-culture system. Human corneal epithelium regenerated on 3T3 fibroblast feeder layer preserved more stem/progenitor cells than explant cultures [20], [22], [23].

However, human epithelia generated on mouse 3T3 fibroblasts may bear a risk of transmitting pathogens and as a consequence their use is not permitted by US FDA for cultivating corneal epithelia for human transplantation. To develop this powerful co-culture system for clinical use without xeno-components, mouse 3T3 fibroblasts must be replaced by human fibroblasts. Encouraged by reports [24]–[27] that human fibroblasts could replace mouse fibroblasts as feeder layers to support ex vivo expansion of human embryonic stem cells, we hypothesize that human fibroblasts can be used as a feeder layer to promote corneal epithelial regeneration for human transplantation. The present study was attempted to identify stable human fibroblast cell lines that could serve as an alternative feeder layer to mouse 3T3 cells.

## Results

### Identification of Human Fibroblast Cell Lines as a Feeder Layer Supporting Ex-vivo Expansion of Human Corneal Epithelial Cells

To identify whether human fibroblasts can serve as a feed layer to support ex vivo expansion of human corneal epithelial cells, five commercially available human fibroblast cell lines were tested with mouse 3T3 fibroblasts as controls. To serve as a feeder for epithelial regeneration, mouse 3T3 fibroblasts were lethally treated with 5 µg/ml mitomycin C for 2 hours, then trypsinized and plated as single cells at 2×10^4^/cm^2^ in a 35-mm dish or 6-well plates. We have observed that higher doses and longer period of mitomycin C treatment are required for human fibroblasts than mouse 3T3 cells. The treatment with 5 µg/ml mitomycin C for 16 hours was tested to be an optimal condition for these human fibroblasts serving as a feeder layer.

**Figure 1:**
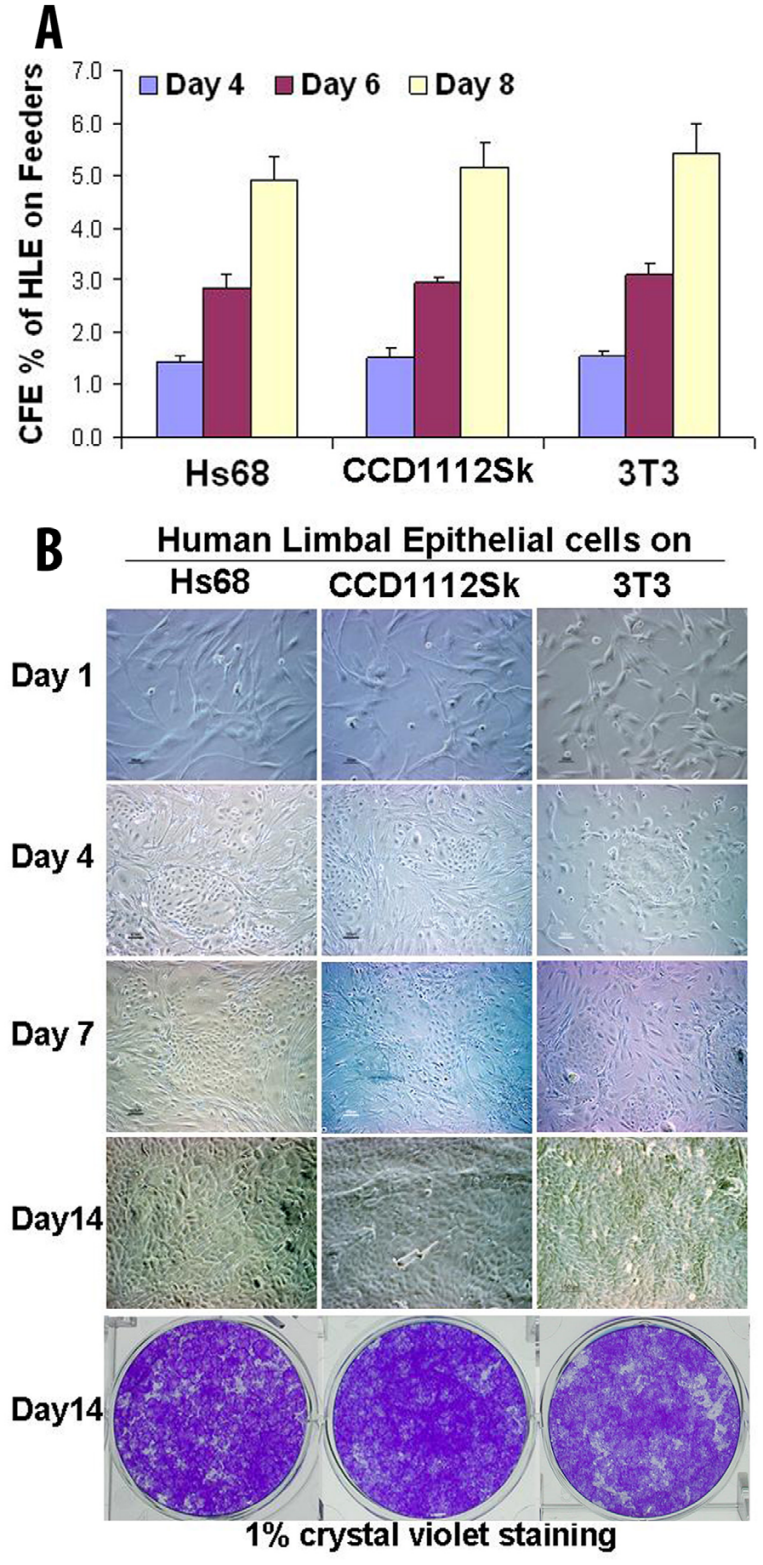
Human limbal epithelial cells (HLE) grown on feeder layers of Hs68, CCD1112Sk or 3T3 fibroblasts after seeded at 1×10^3^ cells/cm^2^ in 6-well plate. A. Colony forming efficiency (CFE) of HLE on three fibroblasts in days 4–8. B. Phase images showing a comparative efficiency of Hs68, CCD1112Sk and 3T3 fibroblasts as a feeder layer in supporting clonogenicity and growth capacity of HLE on days 1–14.

Human corneal epithelial cells were isolated from donor corneal limbus, and seeded at 1×10^3^ cells/cm^2^ on these human fibroblast feeder layers with mouse 3T3 cells as controls [22]. We have observed that human corneal epithelial cells formed fewer colonies and grew slowly on the feeders prepared from human fetus skin fibroblasts Detroit 551 and two newborn foreskin fibroblasts (Hs27 and BJ), when compared with that on a 3T3 feeder layer. Interestingly, two newborn foreskin fibroblasts, Hs68 and CCD1112Sk, displayed a comparative capacity to mouse 3T3 fibroblast feeder layer in supporting human corneal epithelial growth. Tested for 10 passages at 16–25 and 6–15 respectively, Hs68 and CCD1112Sk fibroblasts showed strong support for colony forming efficiency (CFE) and growth capacity of human corneal epithelial cells. As shown in Fig 1A, the CFE was 1.43±0.13% at day 4 and 4.91±0.47% at day 8 on Hs68 fibroblasts, and 1.52±0.17% at day 4 and 5.16±0.47% at day 8 on CCD1112Sk fibroblasts, similar to the CFE on mouse 3T3 feeder layer, 1.56±0.07% at day 4 and 5.41±0.57% at day 8, respectively. The corneal epithelial cells grew rapidly from clonal colonies to confluence in 35-mm dishes (about 1.87−2.41×10^6^ cells covering 9.6 cm^2^ area) in 12–14 days on Hs68, CCD1112Sk or 3T3 feeder layers (Fig 1B), representing 187–241 fold expansion with over 7–8 doublings on human feeders, similar to 3T3 feeder layer.

### The Phenotype of Human Corneal Epithelium Regenerated on Human Fibroblast Feeder Layer

**Figure 2:**
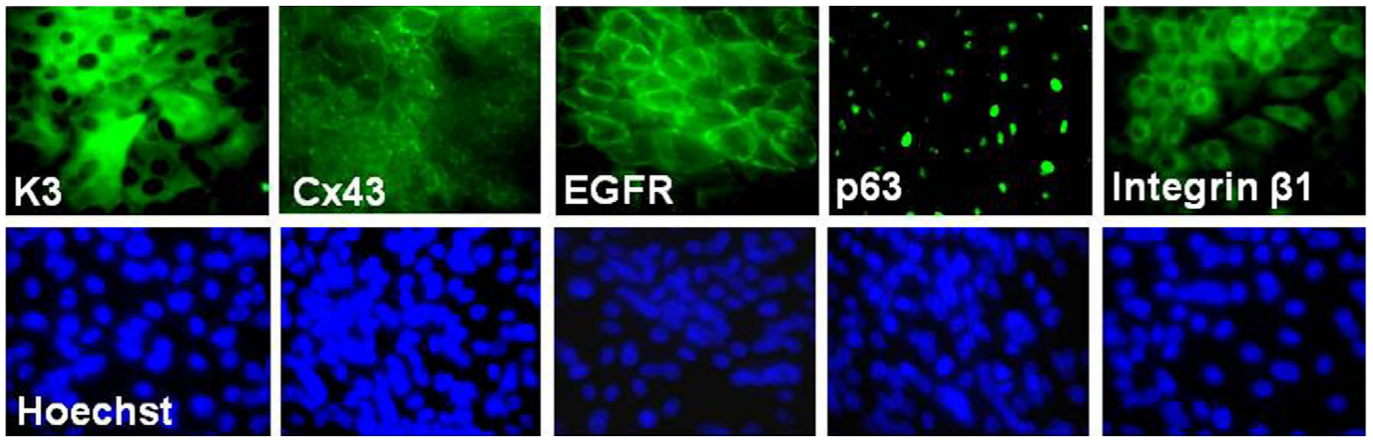
Representative immuno-fluorescent staining profiles for corneal epithelial phenotype. Corneal epithelial markers, including the differentiation markers, keratin 3 (K3), and connexin 43 (Cx43), as well as progenitor markers, EGFR, nuclear p63 and integrin β1, were expressed by HLE regenerated on human feeder of Hs68 fibroblasts; Hoechst 33342 was used for nuclear counterstaining.

**Figure 3:**
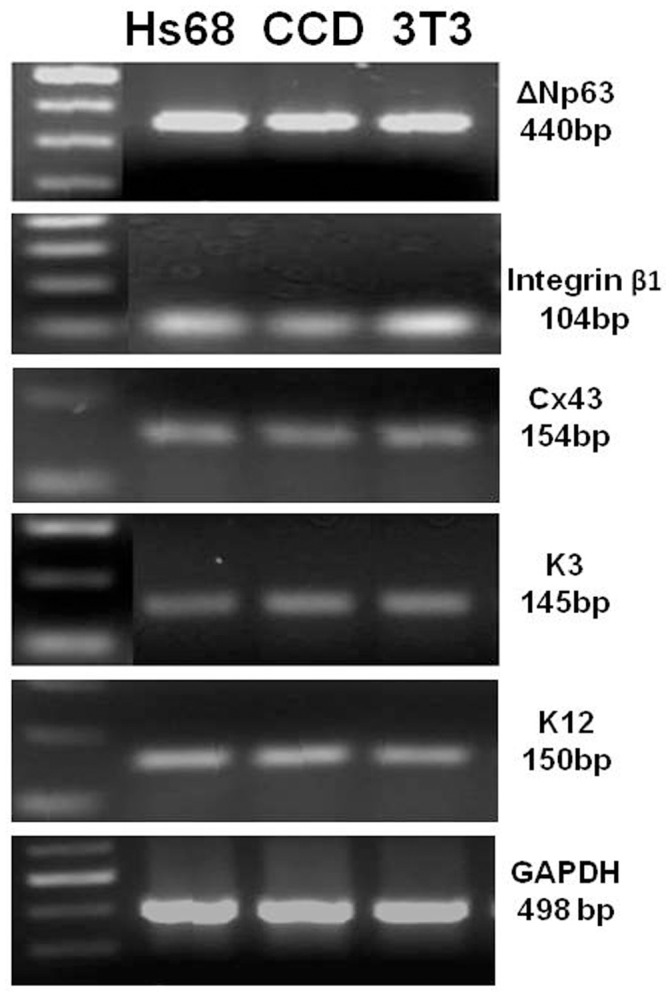
Gene expression profiles for corneal epithelial markers by HLE grown on Hs68, CCD1112Sk (CCD) and 3T3 fibroblasts. Representative RT-PCR images show the mRNA expression of ΔNp63 (440 bp), integrin β1 (104 bp), Cx43 (154 bp), K3 (145 bp) and K12 (150 bp) with GAPDH (498 bp) as internal control. A 100 bp DNA ladder is shown at the left lane.

The phenotype of human corneal epithelium regenerated on Hs68 and CCD1112Sk feeder layers were further characterized by evaluating their morphology and epithelial cell markers. The corneal epithelium generated on these human feeders contained small compact cells and expressed epithelial markers (Fig 2). Immunofluorescent staining revealed that corneal epithelial specific marker cytokeratin (K) 3 was immunolocated in cytoplasm of about 45% cells with relatively large size, which also expressed a differentiation marker gap junction protein connexin 43 (Cx43) at cell membrane. Interestingly, three epithelial progenitor cell markers, nuclear transcription factor p63, integrin β1 and epidermal growth factor receptor (EGFR), immunolocated in the nucleus, cytoplasm or membrane, respectively, were strongly expressed by 22–30% cells with relatively small size. The percentages of positive cells for these markers, characteristics of corneal epithelial progenitor cells, in human corneal epithelia regenerated on human fibroblasts Hs68 and CCD1112Sk were comparable to those on mouse 3T3 feeder layer, as shown in Table 1. To confirm these markers at transcription levels, we further compared their mRNA expression by these corneal epithelial cells generated on human fibroblasts (Hs68 and CCD1112Sk) and 3T3 cells. As shown in Fig 3 evaluated by RT-PCR, the cells grown on these two human feeders expressed typical corneal epithelial markers, including progenitor cell markers, ΔNp63 and integrin β1, and differentiation markers, Cx43, K3 and K12, which was similar to the expression profile of the cells grown on 3T3 mouse feeder. These results indicated that the epithelium regenerated on human feeder layer resembled the phenotype of human corneal epithelium and contained undifferentiated progenitor cells [22], which are important for tissue engineering in regenerative medicine.

**Table 1:** Properties of human corneal epithelia co-cultured on human (Hs58 and CCD1112Sk) and mouse (3T3) fibroblasts.

Fibroblast Feeder	Human Corneal Epithelium*		
	Hs68	CCD1112Sk	3T3
**Proliferation**			
CFE % on Day 4	1.43±0.13	1.52±0.17	1.56±0.07
CFE % on Day 8	4.91±0.47	5.16±0.47	5.41±0.57
Cells at confluence (x10^6^)	2.3±0.18	2.1±0.36	2.4±0.21
Confluence at days	12–14	12–14	12–14
Doubling time (hours)	42.8	43.9	42.4
**Epithelial Markers**			
K3+ Cells%	44.2±9.3	45.3±11.2	42.3±10.4
K14+ Cells%	50.7±13.3	52.27±15.1	48.7±12.3
Cx43+ Cells%	55.5±14.8	57.4±13.5	59.3±15.2
EGFR+ Cells%	27.1±4.4	26.3±6.7	24.3±5.9
Nuclear p63+ Cells%	24.6±5.8	22.3±5.4	23.9±4.7
Integrin β1+ Cells%	28.1±5.7	29.9±6.8	26.3±5.4

* Initiated from 1×10^4^ limbal epithelial cells/35 mm dish (9.6 cm^2^ area).

### Identification of Potential Stroma-derived Factors from the Supportive Human Fibroblasts that Promoted Corneal Epithelial Regeneration

Mouse 3T3 fibroblast feeder layer has been widely used for long-term growth and serial propagation of many types of epithelial cells since 1975 [13], however, the exact identity of this fibroblast-derived supporting activity remains to be elucidated. While human foreskin fibroblast cell lines have been identified to be capable of supporting the corneal epithelial regeneration, we further attempted to identify stroma-derived factors from these supportive fibroblasts. We performed cDNA microarray using Agilent GeneChip to characterize the expression profile and molecular changes of supportive human fibroblasts (Hs68) before and after mitomycin C treatment.

Among 22575 transcripts on a chip, 3400 transcripts, accounting for 15.1% of expressed genes, were regulated after treatment with mitomycin C (5 µg/ml for 16 hours). Among 3,400 changed genes, 202 genes were up-regulated more than 2 fold with *p* value <0.05; while 331 genes were down-regulated more than 50% with *p* value <0.05. The best 3 up-regulated genes were identified: CDKN1A (cyclin-dependent kinase inhibitor 1A, p21, Cip1), GDF15 (growth differentiation factor 15), and FDXR (ferredoxin reductase). The best 10 down-regulated genes were also identified: HIST1H4C (histone cluster 1, H4c), TNFRSF11B (tumor necrosis factor receptor superfamily, member 11b), ITGB1 (integrin, beta 1), COL6A3 (collagen type VI, alpha 3), TWIST2 (twist homolog 2), SMARCD3 (SWI/SNF related, matrix associated, actin dependent regulator of chromatin, subfamily d, member 3), FN1 (fibronectin 1), THBS2 (thrombospondin 2), PTX3 (pentraxin 3, long), and CDK2 (cyclin-dependent kinase 2). Functional pathway analysis revealed that there were 7 genes down-regulated (>2-fold) in 20 genes of the cell proliferation regulation group (data not shown). These genes and related signaling pathways might be potential targets, and further investigations are important for validation and functional identification of stroma-derived genes that promote proliferation and regeneration of human corneal epithelial cells.

## Discussion

Mouse 3T3 fibroblasts have been used as a feeder layer for more than three decades [13] and proven to be the most powerful system among all epithelial culture methods. To avoid xeno-components per clinical use, the mouse 3T3 fibroblasts need to be replaced by human feeders. In the present study, we have evaluated five commercial human fibroblast cell lines, and successfully identified two cell lines of human newborn foreskin fibroblasts, Hs68 and CCD1112Sk, which showed comparative efficiency to a 3T3 feeder layer in supporting human corneal epithelial cell growth and regeneration.

Based on evaluation of epithelial clonal colony forming efficiency and epithelial regeneration capacity, human newborn foreskin fibroblasts, Hs68 and CCD112Sk, showed strong supportive capacity, similar to the mouse 3T3 feeder layer (Fig 1). The seeded epithelial cells (1×10^4^ cells) grew rapidly from colonies to confluence reaching about 1.87−2.41×10^6^ cells in 12–14 days, which represented 187–241 fold expansion with over 7–8 doublings. Cell morphology and immunofluorescent staining further revealed that the regenerated epithelium on these human feeders resembled the phenotype of human corneal epithelium containing undifferentiated progenitor cells. As shown in Fig 2 and 3, the regenerated epithelium expressed three progenitor cell markers, p63, integrin β1 and EGFR, in addition to corneal epithelial specific and differentiated markers K3, K12 and Cx43. The cells expressing the progenitor cell markers reached 22–30%, consistent to our previous report [22].

Signals transmitted from mesenchyme to epithelia or vice versa constitute the basis of reciprocal mesenchymal-epithelial interactions, which are thought to play an essential role in organogenesis and tissue regeneration. In adult tissues, mesenchymal-epithelial cell communications maintain tissue homeostasis and play a key role in epithelial repair [10], [28], [29]. Mesenchymal-epithelial interactions have been successfully applied to in vitro cell culture systems for growing epithelial cells that were difficult to cultivate and serially propagate without the presence of fibroblasts or their products. In the presence of a fibroblast feeder layer, epithelial cells expand rapidly and maintain their progenitor phenotype, while in the absence of fibroblasts, epithelial cells undergo terminal differentiation. After identification of human fibroblast cell lines, Hs68 and CCD112Sk, supporting corneal epithelial regeneration, we performed cDNA microarray to search stroma-derived factors from these supportive fibroblasts. Interestingly, three up-regulated genes and 10 down-regulated genes have been identified to be involved in the functions of fibroblast feeders. Functional pathway analysis using GenMAPP 2.0 and MAPPFinder further revealed that 7 genes were down-regulated in 20 genes of cell proliferation regulation group. These genes and related pathways are potential targets that are useful for further validation and functional identification of the stroma-derived genes that promote proliferation of limbal stem cells.

In conclusion, we have identified two commercially available cell lines of human newborn foreskin fibroblasts, Hs68 and CCD112Sk, which support human corneal epithelial regeneration. The cultivated corneal epithelium on human fibroblast feeder layers resembles the phenotypes of human corneal epithelium containing progenitor cells. The development of human fibroblast feeders will represent a major advance in the field of corneal epithelial regeneration. Human feeders have potential in use for tissue bioengineering, not only for corneal epithelium, but also possibly for other epithelial tissues. In particular, this new technique would greatly promote corneal tissue bioengineering in regenerative medicine to improve the treatment for patients with blinding corneal disease.

## Materials and Methods

### Preparation of Fibroblasts as a Feeder Layer

Mouse 3T3 embryo fibroblasts (CCL-92) and five human fibroblast cell lines were purchased from ATCC (Manassas, VA), including fetus skin fibroblasts (Detroit 551, CCL-110), and 4 newborn foreskin fibroblasts, Hs27 (CRL-1634), Hs68 (CRL-1635), CCD1112Sk (CRL-2429) and BJ (CRL-2522). All these fibroblasts were cultured in DMEM containing 10% FBS, and subcultured with 1∶4 split for several passages when they reached 80–90% confluence The confluent fibroblasts were lethally treated with mitomycin C at 5 µg/ml for different time periods (2 hours for 3T3 cells, and 16 hours for human fibroblasts), and then trypsinized and plated at 2×10^4^ cells/cm^2^ in 35-mm dishes or 6-well culture plates as feeder layers used for corneal epithelial cell culture.

### Isolation of Limbal Epithelial Single Cells for Ex vivo Expansion in Co-cultures

Cadaveric human corneoscleral tissues were obtained from the Lions Eye Bank of Texas (LEBT, Houston, TX). Single epithelial cells were isolated from corneal limbal tissues as previously described [22], [30], [31]. In brief, the limbal rims were incubated with 5 mg/ml of dispase II (Roche, Indianapolis, IN) at 37°C for 2 hours. The detached limbal epithelial sheets were collected and incubated with 0.05% trypsin/0.03% EDTA at 37°C for 5–10 minutes to isolate single cells. Single cells of human limbal epithelium were then seeded at 1×10^3^ cells/cm^2^ on different fibroblast feeders in a supplemented hormonal epidermal medium (SHEM) containing 5% FBS to evaluate the colony forming efficiency, growth capacity and phenotypes of cultivated corneal epithelium [22].

### Assays for Clonogenicity and Proliferative Potential

Limbal epithelial single cells were seeded, in triplicate, at 1×10^3^ cells/cm^2^ on a feeder layer (human or mouse fibroblast) in 6-well culture plates [13], [22], [30]. The colony forming efficiency is calculated as a percentage of the number of colonies at days 4–8 generated by the number of epithelial cells plated in a well. The clonal growth capacity was evaluated on days 12–14 when the cultures were stained with 1% crystal violet.

### Immunofluorescent and Immunohistochemical Staining

Cell cultures were fixed in cold methanol or 2–4% paraformaldehyde and permeabilized with 0.2% Triton X-100 in PBS for 10 minutes each. Immunofluorescent staining were performed with our previous methods [22], [28], [32], [33] using primary antibodies against p63 [34], integrin β1, EGFR, K14, K3, connexin 43 (Calbiochem, Labvision, ICN Pharmaceuticals, Santa Cruz Biotechnology, Chemicon International, or Invitrogen, respectively), with Alexa Fluor 488 conjugated secondary antibodies (Invitrogen), and counter-staining with Hoechst 33342 (Sigma). Specimens without primary antibody or with IgG isotype were used as negative controls. The staining were evaluated and photographed with a Nikon Eclipse microscope.

### Total RNA Extraction and Reverse Transcription Polymerase Chain Reaction (RT-PCR)

Total RNA was isolated from cells using a Qiagen RNeasy® Mini kit according to manufacturer’s protocol. The RNA samples were stored at –80°C before use. The RNA quality and concentration were measured by Agilent 2100 Bioanalyzer and NanoDrop® ND-1000 Spectrophotometer. The mRNA expression of different molecular markers was analyzed by RT-PCR as previously described [33], [35]. The specific primer pairs for each gene were designed from published human gene sequences (Table 2).

**Table 2:** Human primer sequences used for RT-PCR.

Gene	Accession	Sense primer	Antisense primer	PCR Product
▵Np63	XM_036421	CAGACTCAATTTAGTGAG	AGCTCATGGTTGGGGCAC	440 bp
Integrin β1	X_07979	AGTGAATGGGAACAACGAGGTC	CAATTCCAGCAACCACACCA	104 bp
Cx43	M_65188	CCTTCTTGCTGATCCAGTGGTAC	ACCAAGGACACCACCAGCAT	154 bp
K12	D78367	ACATGAAGAAGAACCACGAGGATG	TCTGCTCAGCGATGGTTTCA	150 bp
K3	NM_057808	GGCAGAGATCGAGGGTGTC	GTCATCCTTCGCCTGCTGTAG	145 bp
GAPDH	M_33197	GCCAAGGTCATCCATGACAAC	GTCCACCACCCTGTTGCTGTA	498 bp

### Agilent Human cDNA Microarray Analysis

Agilent Human cDNA Microarray (Agilent Technologies, Santa Clara CA) assays were performed by the Microarray Core Facility at Baylor College of Medicine. Total RNA (15 µg) of human fibroblast cell line Hs68 (CRL-1635) without or with mitomycin C treatment was converted into cDNA by reverse transcription and labeled with green Cye3- (for control) or red cye5- (for treated) dUTP. An array chip was hybridized with labeled cDNAs overnight, scanned with an Axon 4200, and analyzed using the program Acuity from Axon Technologies. Data is viewed as a normalized ratio of Cye5/Cye3. The ratio was indicatives of no changed (Cye5/Cye3 = 1), decreased (Cye5/Cye3<1) or increased (Cye5/Cye3>1) levels of gene expression relative to the control sample. Two- or more fold (Log Ratio of Cye5/Cye3>1 or −1) changed genes were analyzed by multiple software including the Bioconductor suite of R packages in R 1.7.1, (http://www.r-project.org), GenMAPP 2.0 and MAPPFinder (http://www.GenMAPP.org) [36], [37].
